# Supplementary material for: Transposable elements mediate genetic effects altering the expression of nearby genes in colorectal cancer
Source: Nat Commun. 2024 Jan 25;15:749. doi: 10.1038/s41467-023-42405-0 (PMC10811328; doi:10.1038/s41467-023-42405-0)
Supplement: Supplementary file 1 — Supplementary Information [file 41467_2023_42405_MOESM1_ESM.pdf]

Supplementary information

Transposable elements mediate genetic effects altering the expression of nearby genes in colorectal cancer

Nikolaos M. R. Lykoskoufis[1,2,3,4*], Evarist Planet[5], Halit Ongen[1,2,3,**], Didier Trono[5,**], Emmanouil T. Dermitzakis[1,2,3*,**]

[1] Department of Genetic Medicine and Development, University of Geneva Medical School, 1211 Geneva, Switzerland

[2] Institute for Genetics and Genomics in Geneva (iGE3), University of Geneva, 1211 Geneva, Switzerland

[3] Swiss Institute of Bioinformatics, 1211 Geneva, Switzerland

[4] NGS-AI JSR Life Sciences, Route de la Corniche 8, 1066, Epalinges, Switzerland

[5] School of Life Sciences, Ecole Polytechnique Fédérale de Lausanne (EPFL), 1015, Lausanne, Switzerland

* Corresponding authors: N.M.R Lykoskoufis: nikolaos.lykoskoufis@gmail.com and E.T.D: emmanouil.dermitzakis@unige.ch

Department of Genetic Medicine and Development, University of Geneva,

1 rue Michel-Servet, 1211 Geneva, Switzerland

** These authors jointly supervised this work.

Supplementary figures

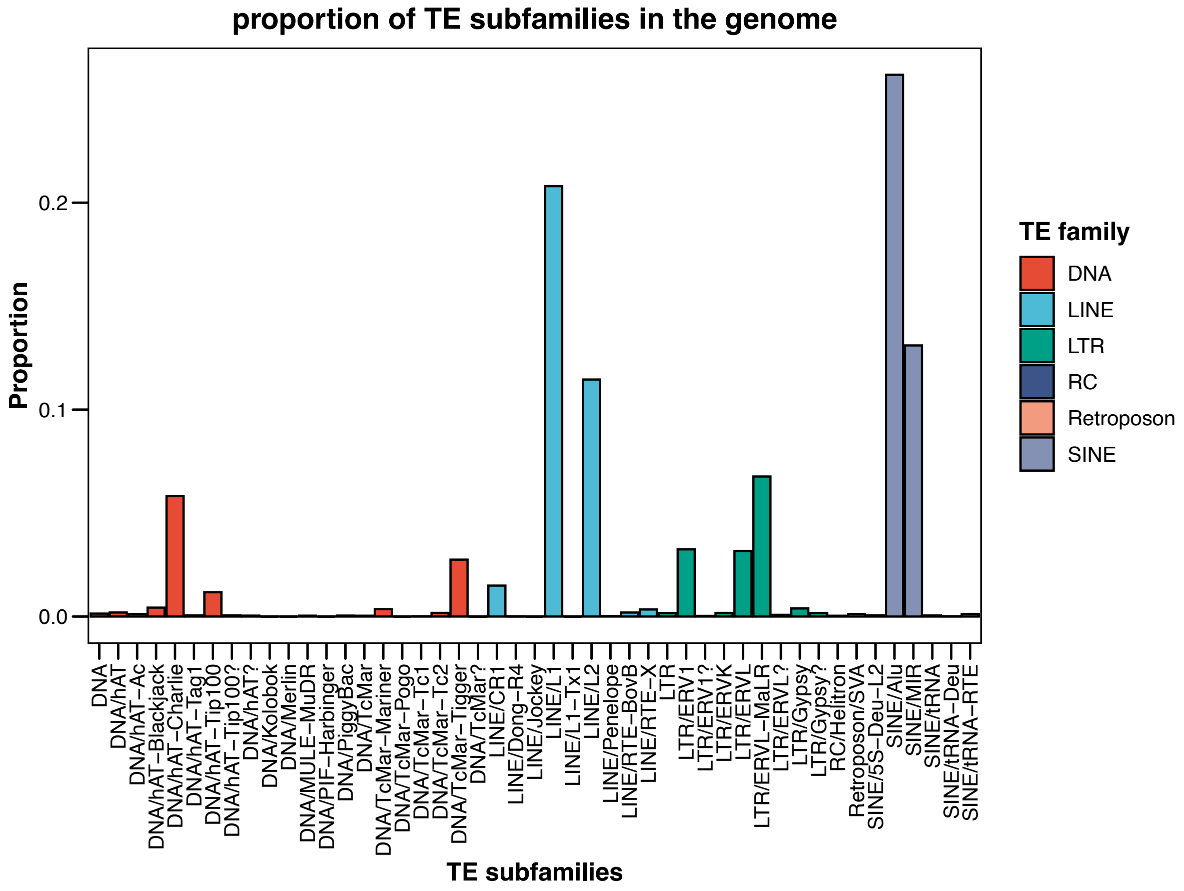

Supplementary figure 1 | *Proportion of TE subfamilies in the human genome*. The majority of transposable elements in the human genome are Alu and tRNA from the SINE family, followed by L1 and L2 elements from the LINE family and ERV1, ERVL and ERVL-MaLR from the LTR family. Source data are provided as a Source Data file.

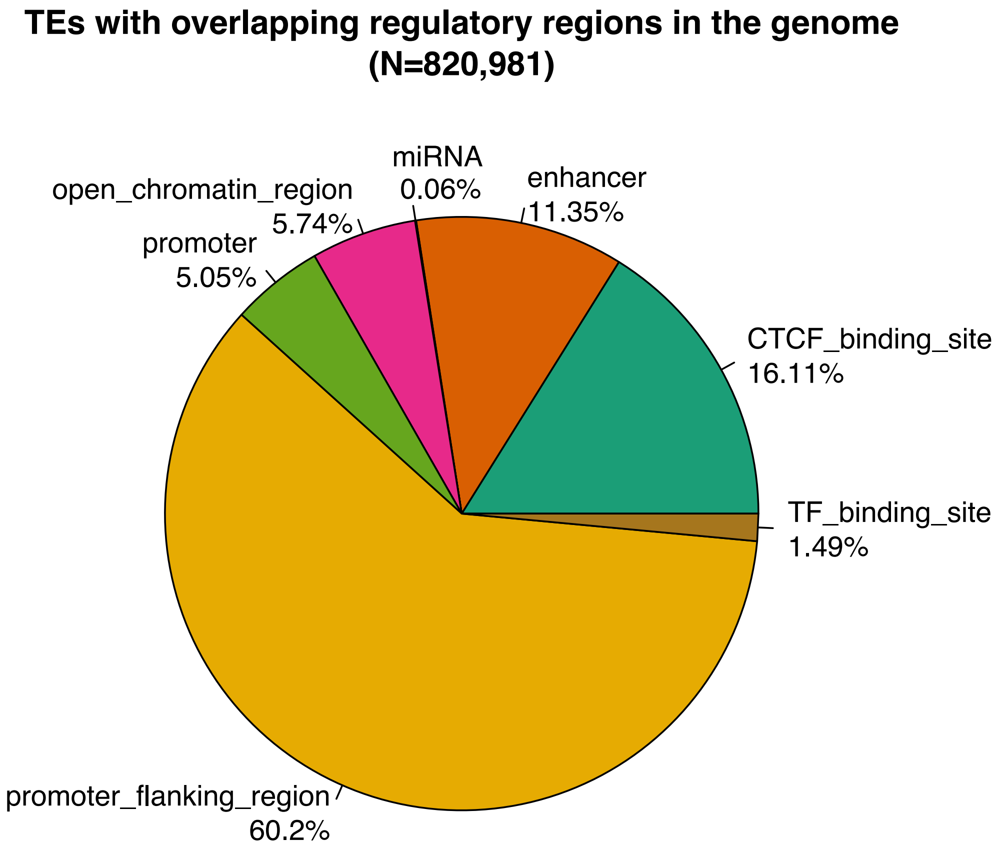

Supplementary Figure 2 | TEs overlapping regulatory regions in the human genome. Pie plot representing the proportion of transposable elements overlapping with each regulatory regions. Of the ~4.6 million TEs in the human genome, 820,981 TEs are overlapping with at least one regulatory region. Source data are provided as a Source Data file.

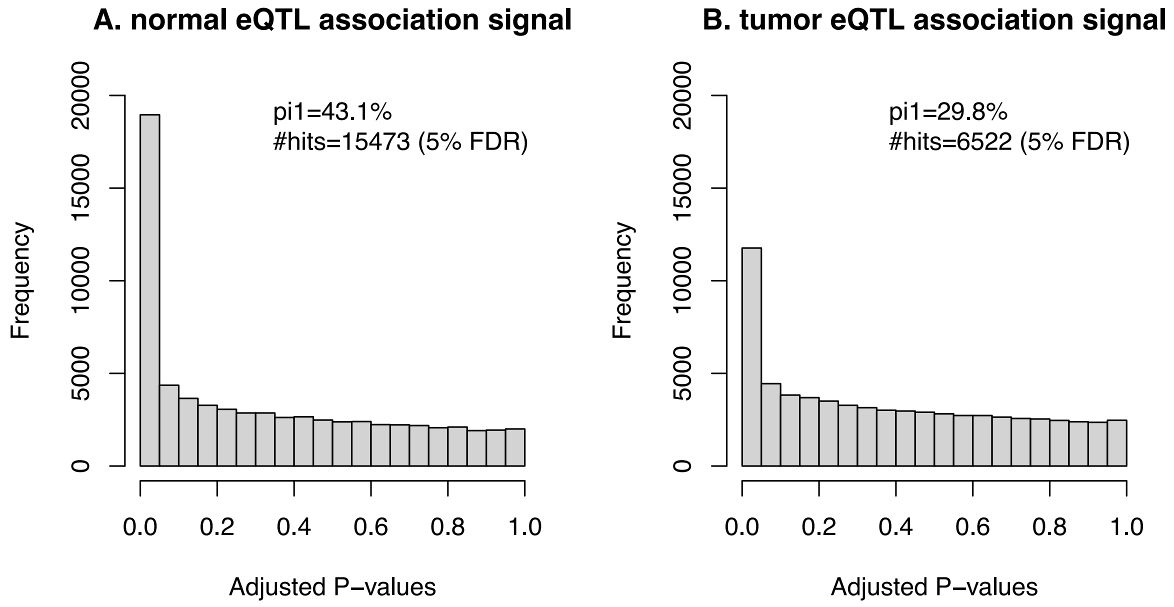

Supplementary figure 3 | *cis eQTL discovery p-value distribution*. Histograms of p-value distribution of *cis*- eQTL discovery in (**A**) normal and (**B**) tumor. We observe a higher pi1 in normal (two-sided pi1 = 43.1%) compared to tumor (two sided pi1 = 29.8%). Source data are provided as a Source Data file. Source data are provided as a Source Data file.

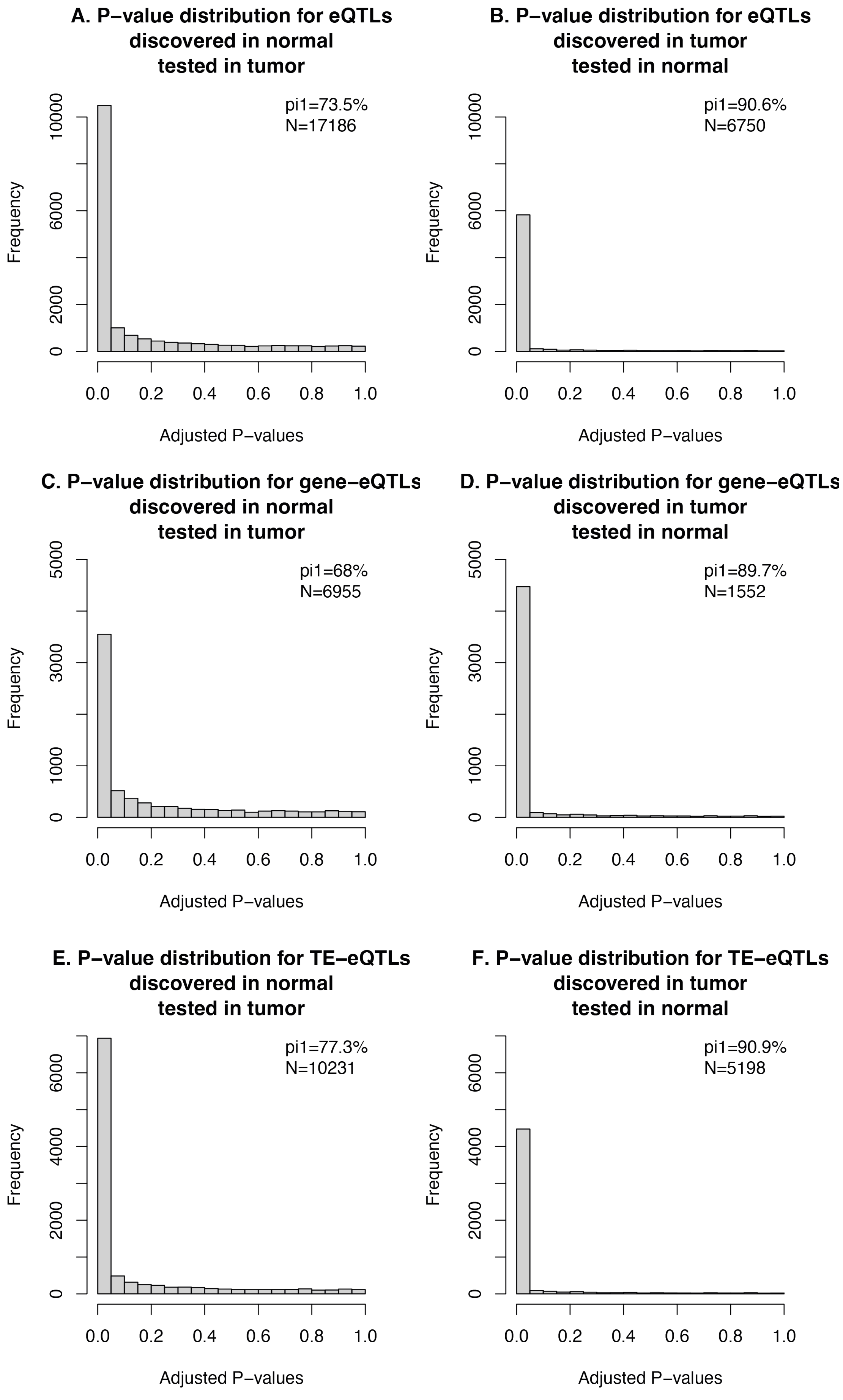
A. P−value distribution for eQTLs discovered in normal tested in tumor
pi1=73.5%
N=17186
B. P−value distribution for eQTLs discovered in tumor tested in normal
pi1=90.6%
N=6750
C. P−value distribution for gene−eQTLs discovered in normal tested in tumor
pi1=68%
N=6955
D. P−value distribution for gene−eQTLs discovered in tumor tested in normal
pi1=89.7%
N=1552
E. P−value distribution for TE−eQTLs discovered in normal tested in tumor
pi1=77.3%
N=10231
F. P−value distribution for TE−eQTLs discovered in tumor tested in normal
pi1=90.9%
N=5198
Frequency
Adjusted P−values
0.0
0.2
0.4
0.6
0.8
1.0

Supplementary figure 4 | P-value distributions of significant SNP-gene or SNP-TE pairs tested in the other tissue. The pi1 (two-sided) statistic estimates the tissue sharing of eQTLs by estimating the proportion of significant hits. (**A**) represents the p-value distribution for TE and gene-eQTLs discovered in normal taken together and tested in tumor. (**B**) represents the p-value distribution of all TE and gene-eQTLs discovered in tumor and tested in normal. (**C**) represents the p-value distribution for gene-eQTLs discovered in normal and tested in tumor. (**D**) represents the p-value distribution for gene-eQTLs discovered in tumor and tested in normal. (**E**) represents the p-value distribution for TE-eQTLs discovered in normal and tested in tumor. (**F**) represents the p-value distribution for TE-eQTLs discovered in tumor and tested in normal. Effectively, for all eQTLs, we took the SNP-TE or SNP-gene pairs discovered in normal and tested them in tumor and vice-versa. We observe that SNP-TE and SNP-gene pairs discovered in tumor are replicated better in normal than normal eQTLs tested in tumor. Source data are provided as a Source Data file.

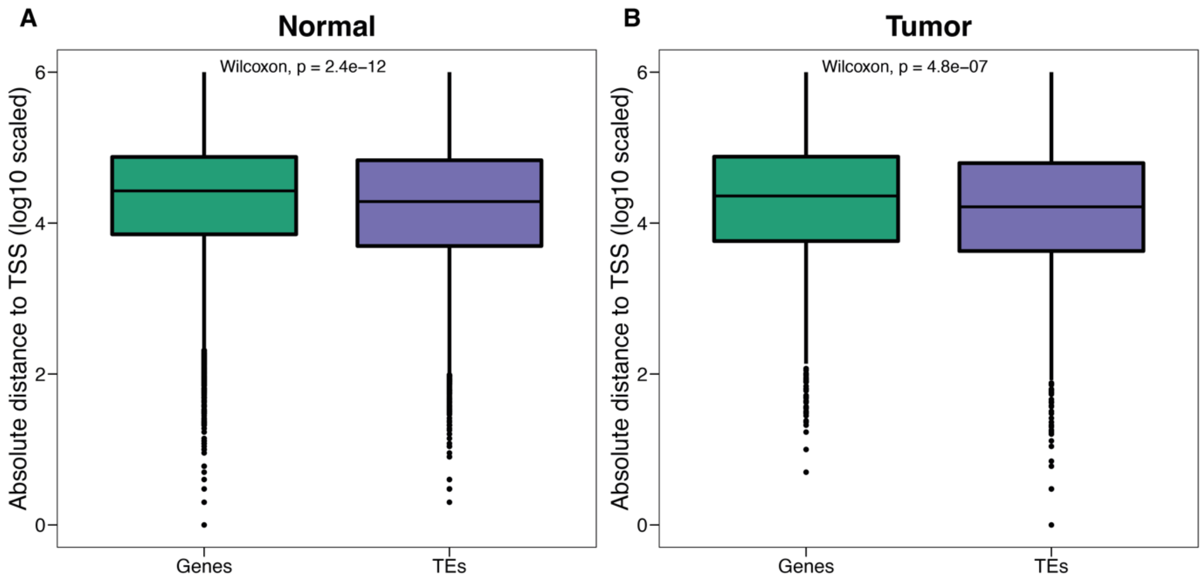

Supplementary figure 5 | TE and gene eQTL distance to TSS in normal and tumor. (A) represents the absolute distance of eQTL variants to their associated genes (green) or TEs (purple) in normal (Gene eQTLs boxplot values: minima = 0; 1st quartile = 3.851; median = 4.427; mean = 0; 3rd quartile = 4.875; maxima = 5.99; TE-eQTLs boxplot values: minima = 0; 1st quartile = 3.691; median = 4.283; mean = 0; 3rd quartile = 4.831; maxima = 6). (B) represents the absolute distance of eQTL variants to their associated genes or TEs in tumor (Gene eQTLs boxplot values: minima = 0; 1st quartile = 3.853; median = 4.392; mean = 0; 3rd quartile = 4.886; maxima = 5.992; TE-eQTLs boxplot values: minima = 0.60; 1st quartile = 3.76; median = 4.33; mean = 4.26; 3rd quartile = 4.83; maxima = 5.99). We observe that in both normal and tumor, TE-eQTLs are closer to the TSS of TEs than gene-eQTLs are to the TSS of genes with n=5,999 gene eQTLs and n=9,474 TE-eQTLs in normal and n=1,490 gene-eQTLs and n=5,032 TE-eQTLs in tumor (two-sided Wilcoxon p= 2.4e-12 in normal and p= 4.8e-07 in tumor). This could be because of the smaller evolutionary time TEs have in the human genome compared to genes, making local effects much more likely to occur. Source data are provided as a Source Data file.

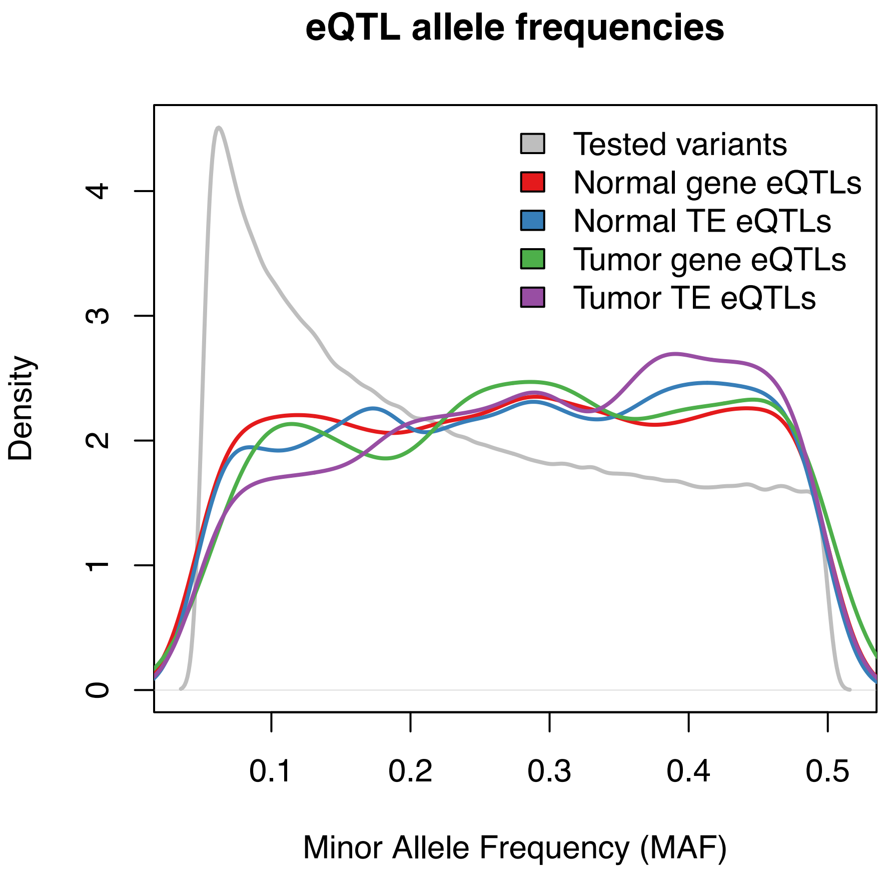

Supplementary figure 6 | eQTL variant allele frequencies. We observe that the allele frequencies for the TE- and gene-eQTLs are very similar in normal and tumor. Source data are provided as a Source Data file.

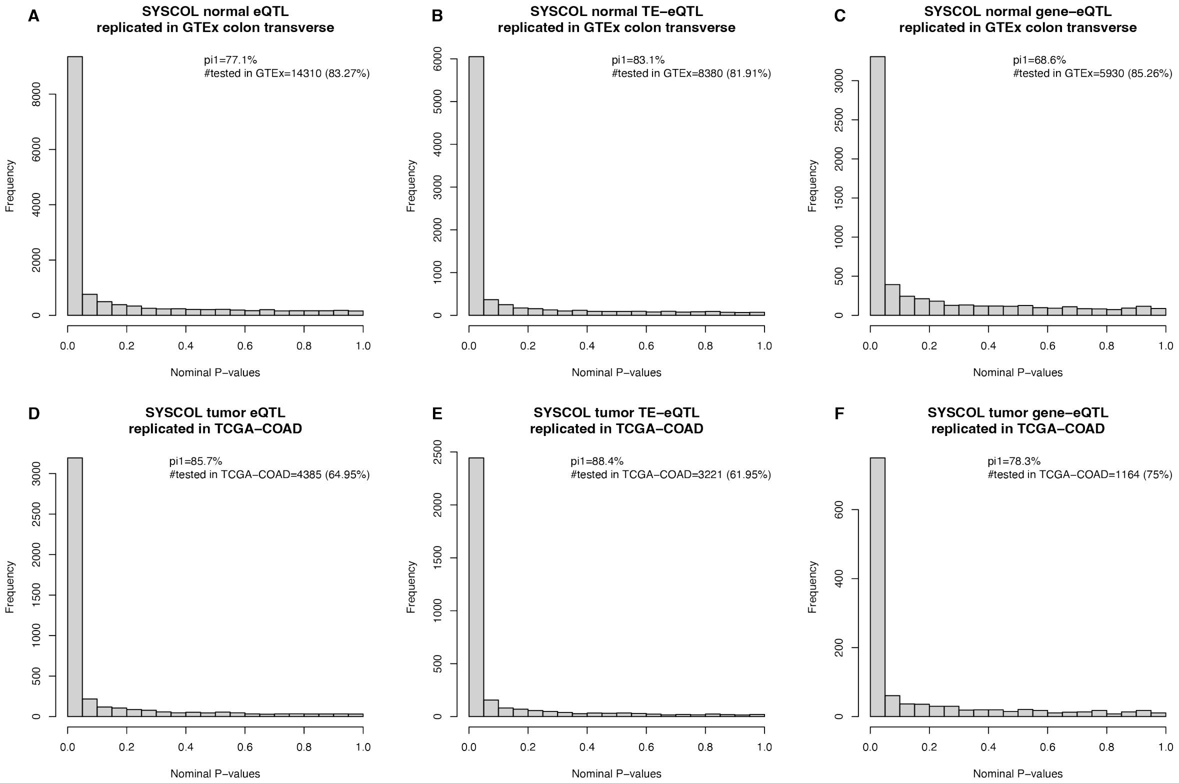

Supplementary figure 7: replication of our eQTL findings in external datasets. Figure representing the p-value distribution of the replication of SYSCOL eQTLs and the two-sided pi1s estimating the proportion of significant hits observed. Panels A to C represent the replications of SYSCOL normal eQTLs in GTEx colon transverse, where **(A)** represents the p-value distribution of gene- and TE-eQTLs taken together, **(B)** the p-value distribution of SYSCOL normal TE-eQTLs and **(C)** the p-value distribution of SYSCOL normal gene-eQTLs. Panels D to F represent the replication of the SYSCOL tumor eQTLs in colon adenocarcinoma (TCGA-COAD), where **(D)** is the p-value distribution of all gene- and TE-eQTLs taken together, **(E)** the p-value distribution of SYSCOL tumor TE-eQTLs and **(F)** the p-value distribution of SYSCOL tumor gene-eQTLs. We observe that in both normal and tumor TE-eQTLs (pi1 normal = 83.1%; pi1 tumor = 88.4%) and gene-eQTLs (pi1 normal = 68.5%; pi1 tumor = 78.3%) have a very high replication in GTEx colon transverse and TCGA-COAD, respectively. Source data are provided as a Source Data file.

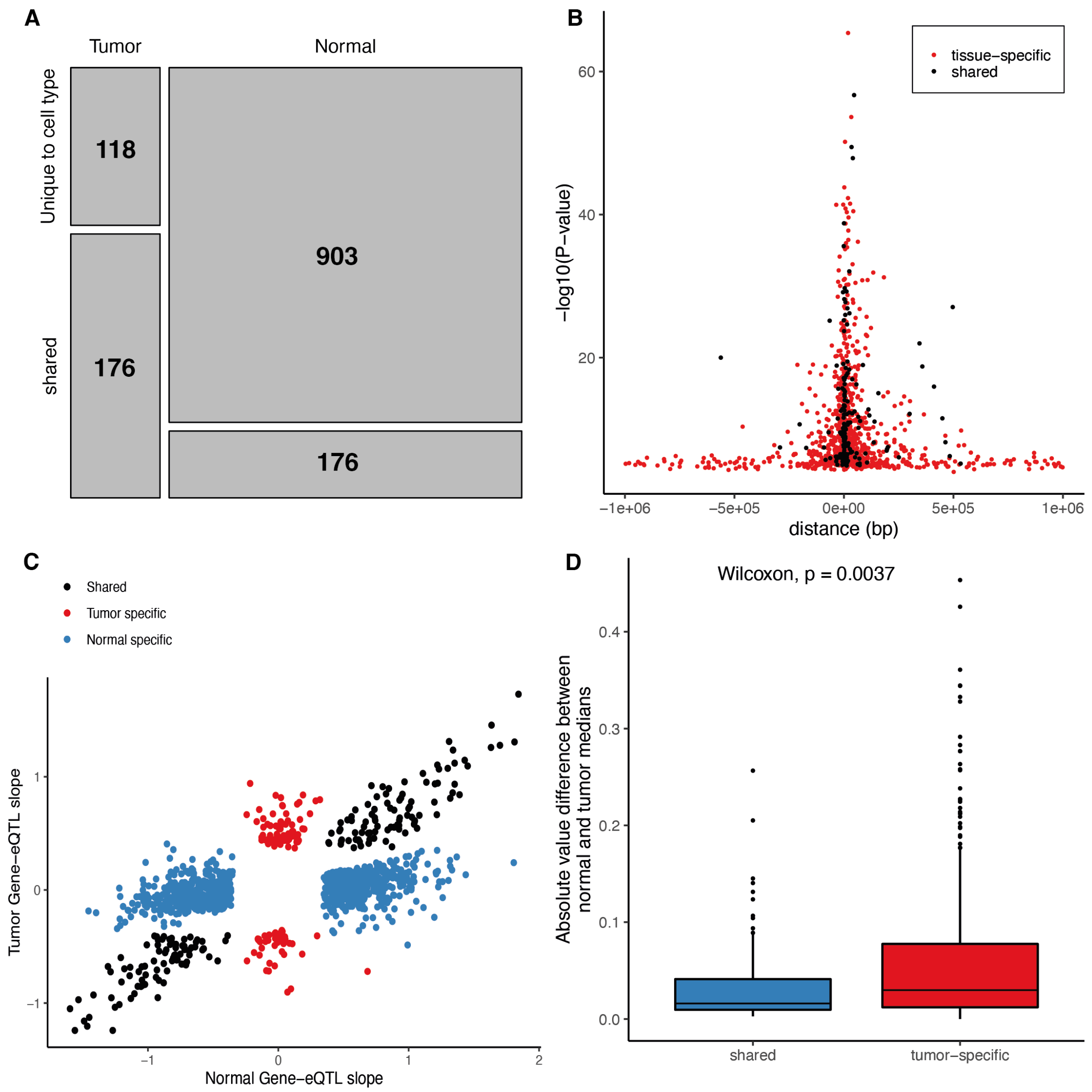

Supplementary figure 8 | Tissue specificity of Gene-eQTLs. (**A**) Mosaic plot of tissue specificity of Gene-eQTLs. (**B**) Tissue specificity and distance of Gene-eQTL to transcription start site (TSS). The shared Gene-eQTLs (black) are closer to the TSS than are the tissue specific Gene-eQTLs (red) (two-sided Wilcoxon P<2.2e-16). (**C**) Gene-eQTL slopes for the normal specific Gene-eQTLs in blue, the tumor specific in red and shared in black. (**D**) Boxplot of the absolute value difference of median methylation betas between normal and tumor samples for shared (n=432) and tumor-specific (n=90) gene-eQTLs. Tumor-specific gene-eQTLs boxplot values: minima = 1.57e-5; 1st quartile = 0.012; median = 0.0298; mean = 0.058; 3rd quartile = 0.077; maxima = 0.454. Shared gene-eQTLs boxplot values: minima = 2.805e-3; 1st quartile = 9.514e-3; median = 0.016; mean = 0.036; 3rd quartile = 0.041; maxima = 0.256. Source data are provided as a Source Data file.

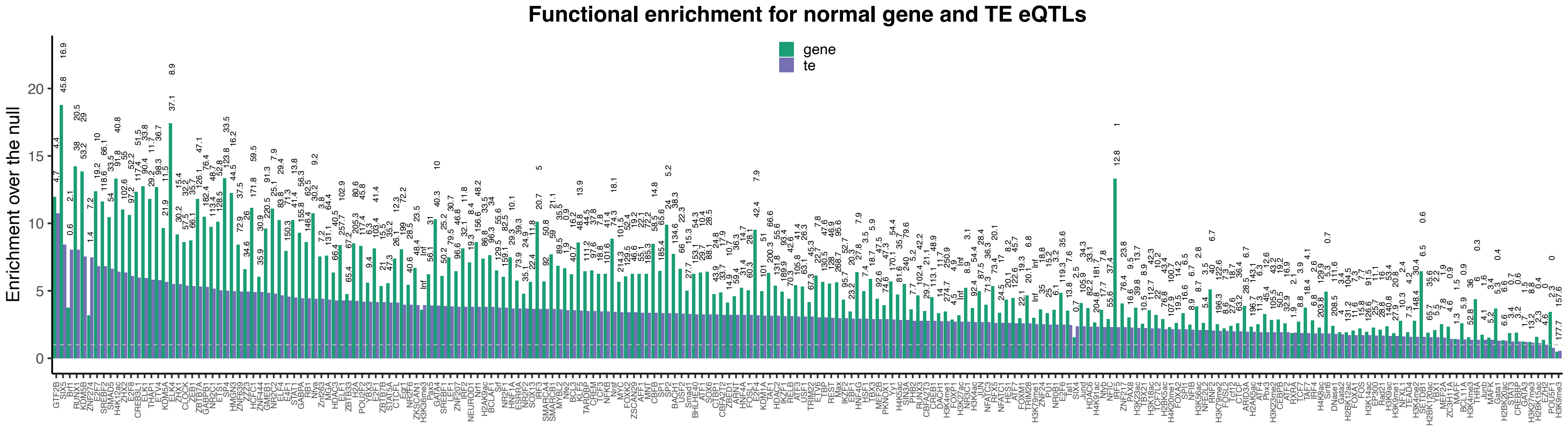

Supplementary Figure 9 | Functional enrichment for gene- and TE-eQTLs in normal. Enrichment over the null for gene- and TE-eQTLs in normal. Only plotting cases where the enrichment was significant at 5% False Discovery Rate (FDR) for either gene- or TE-eQTLs. Source data are provided as a Source Data file.

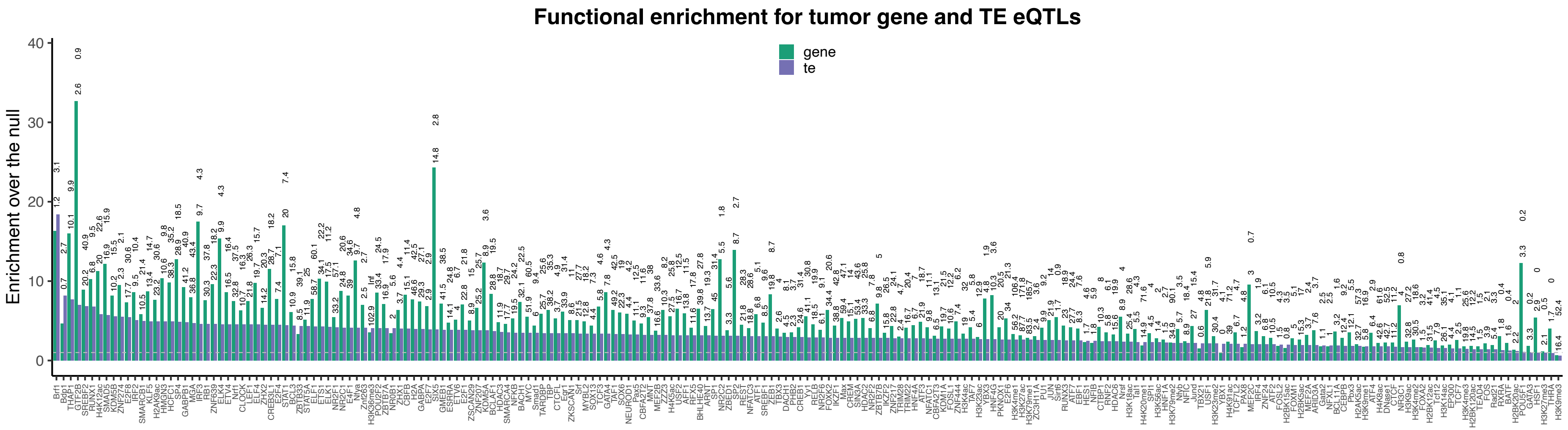

Supplementary Figure 10 | Functional enrichment for gene- and TE-eQTLs in tumor. Enrichment over the null for gene- and TE-eQTLs in tumor. Only plotting cases where the enrichment was significant at 5% False Discovery Rate (FDR) for either gene- or TE-eQTLs. Source data are provided as a Source Data file.

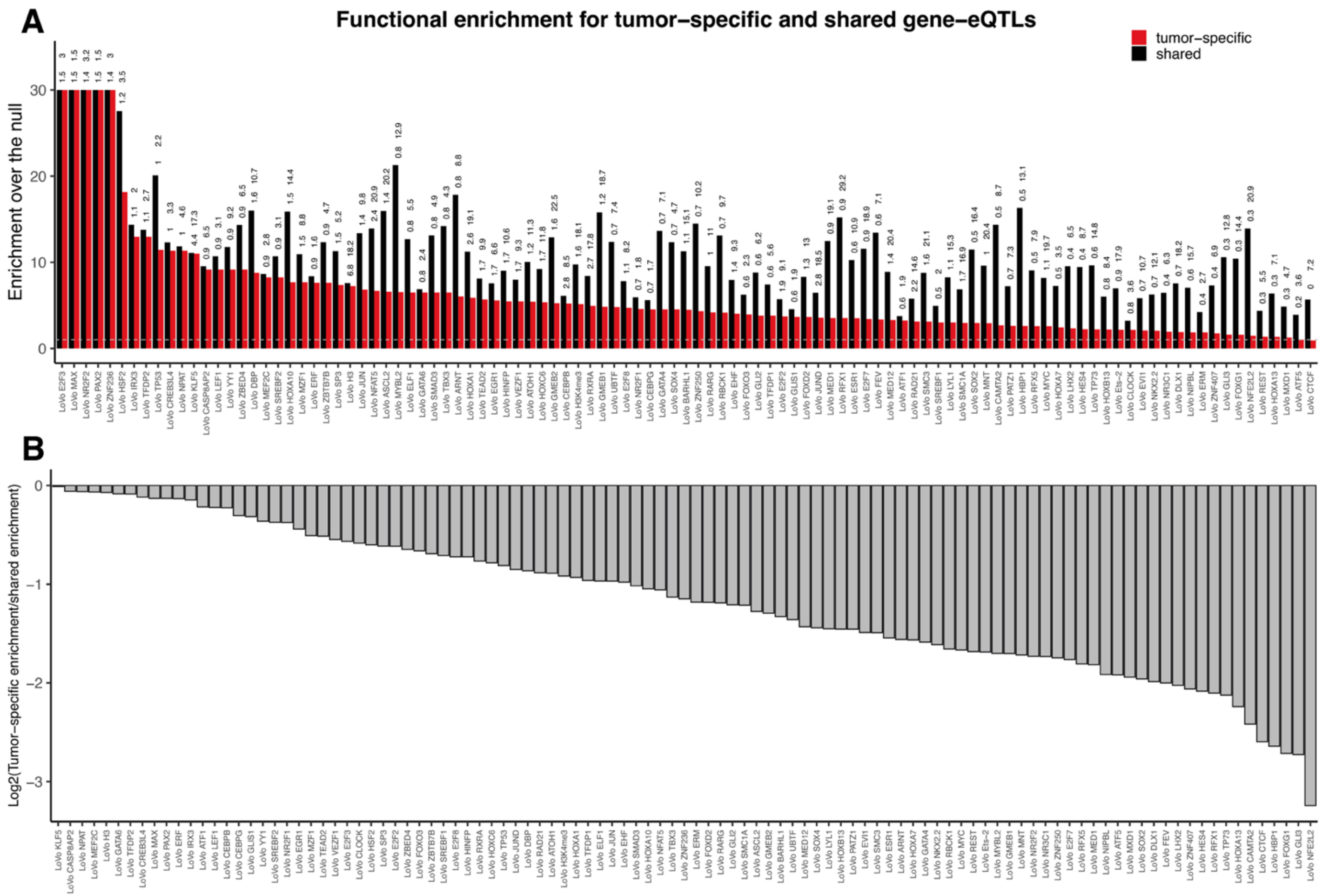

Supplementary figure 11 | Functional enrichment for Tumor-specific and shared gene-eQTLs. (**A**) Enrichment over the null for tumor-specific and shared gene-eQTLs. (**B**) log2 ratio between tumor-specific enrichment and shared enrichment. Only plotting cases where the enrichment was significant at 5% False Discovery Rate (FDR) for either tumor-specific or shared gene-eQTLs. None of the TFs have a stronger enrichment for tumor-specific gene-eQTLs indicating that these TFs regulate gene expression in both the normal and tumor state. Source data are provided as a Source Data file.

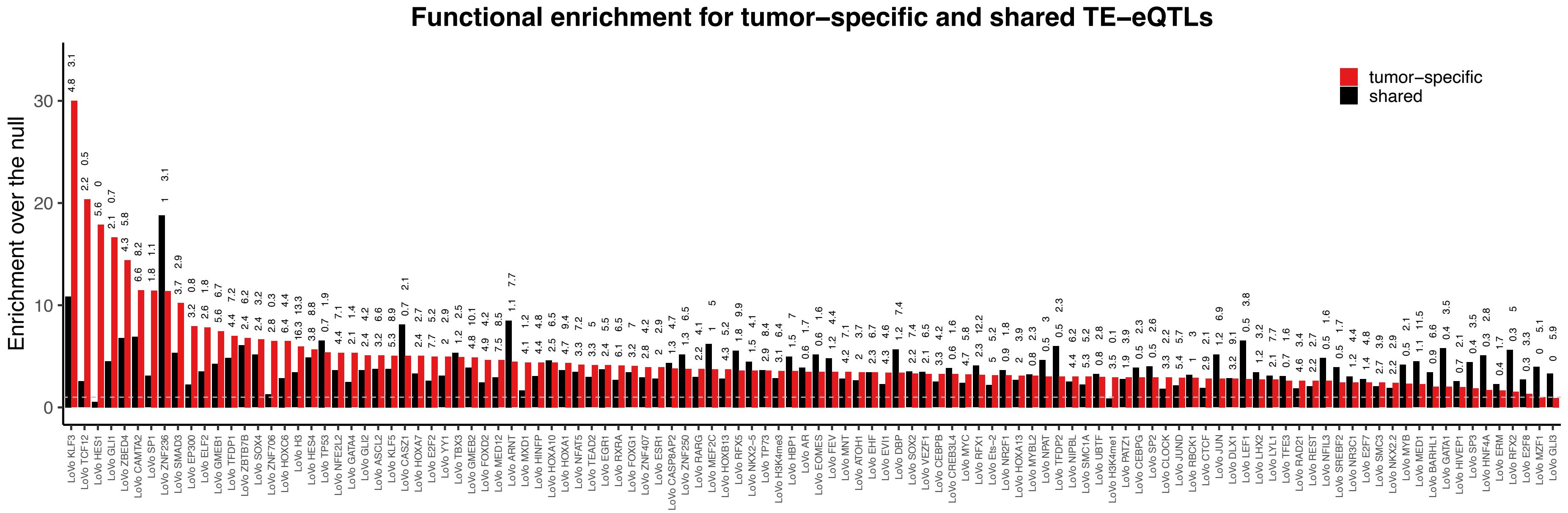

Supplementary figure 12 | Functional enrichment for Tumor-specific and shared TE-eQTLs. Enrichment over the null for tumor-specific and shared TE-eQTLs. Only plotting cases where the enrichment was significant at 5% False Discovery Rate (FDR) for either tumor-specific or shared TE-eQTLs. Source data are provided as a Source Data file.

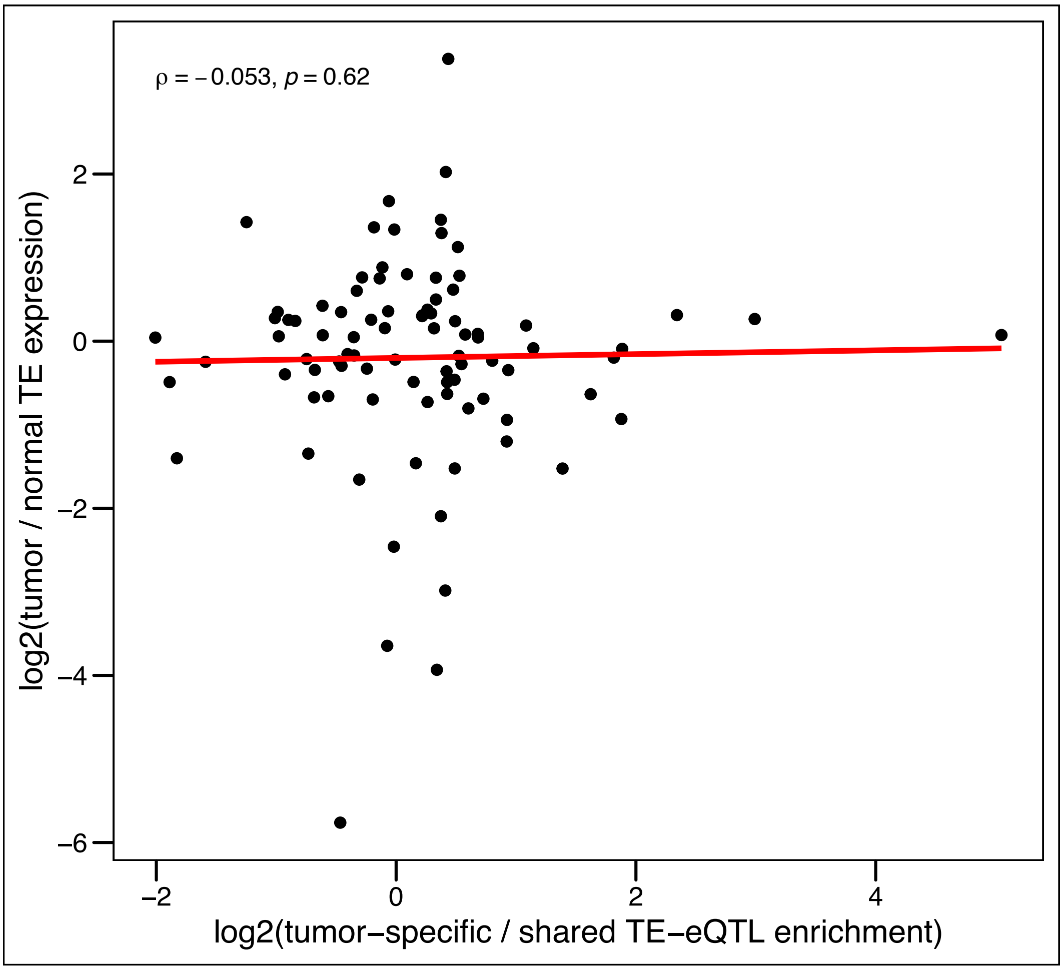

Supplementary figure 13 | Correlation of differential enrichment of functional binding sites and differential expression of the corresponding transcription factors. We compared the ratio of tumor expression over the normal expression of differentially expressed transcription factors to the ratio of enrichment for the binding sites of the same transcription factors in tumor specific TE-eQTLs over the shared TE-eQTLs. We find no significant correlation (two-sided pearson correlation, p = 0.62) indicating that differential expression of the corresponding TFs do not drive the tumor-specific TE-eQTLs. Source data are provided as a Source Data file.

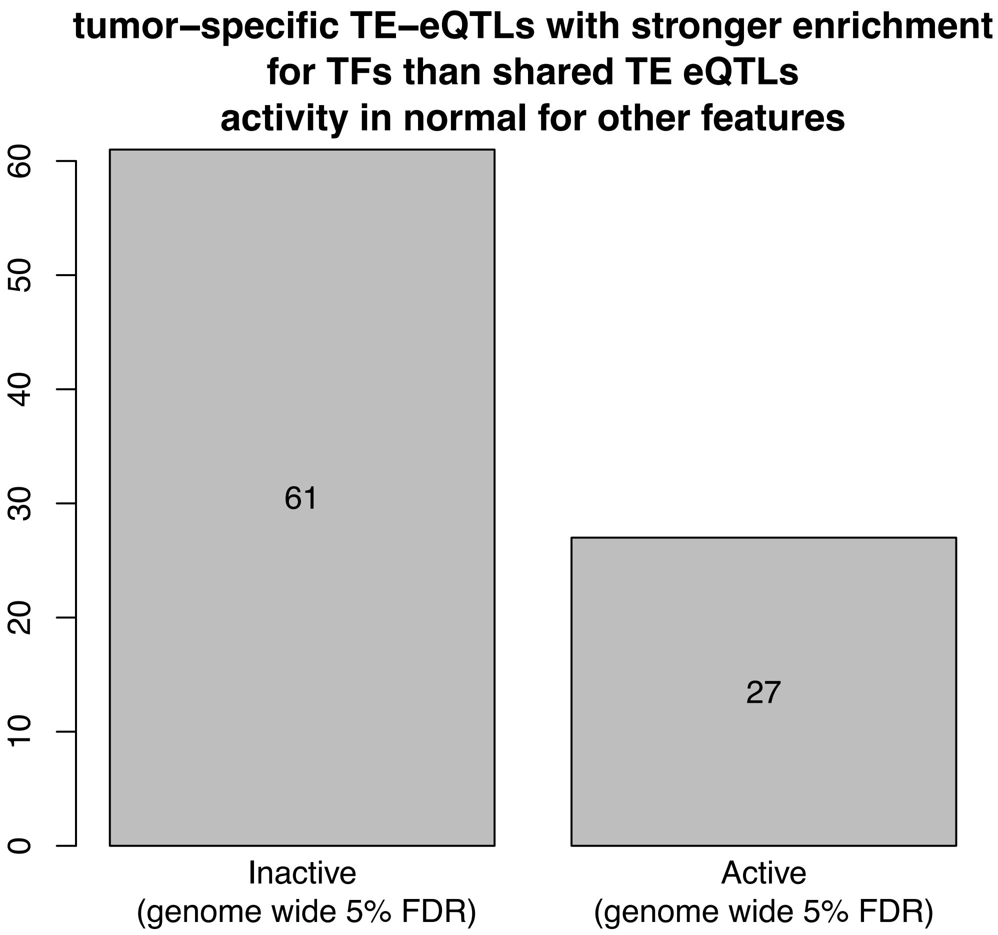

Supplementary figure 14 | Tumor-specific TE-eQTLs overlapping with the enriched transcription factors and their activity in normal regarding other genes/TEs. We checked whether any of the tumor-specific TE-eQTLs overlapping with the transcription factors we found to have a stronger enrichment for tumor-specific TE-eQTLs are active or inactive eQTLs for other TEs or genes in *cis*. We discovered that 61 of them are not significant eQTLs for any TE or gene in normal indicating that these regions are probably inactive and get activated in tumorigenesis. FDR: False Discovery Rate. Source data are provided as a Source Data file.

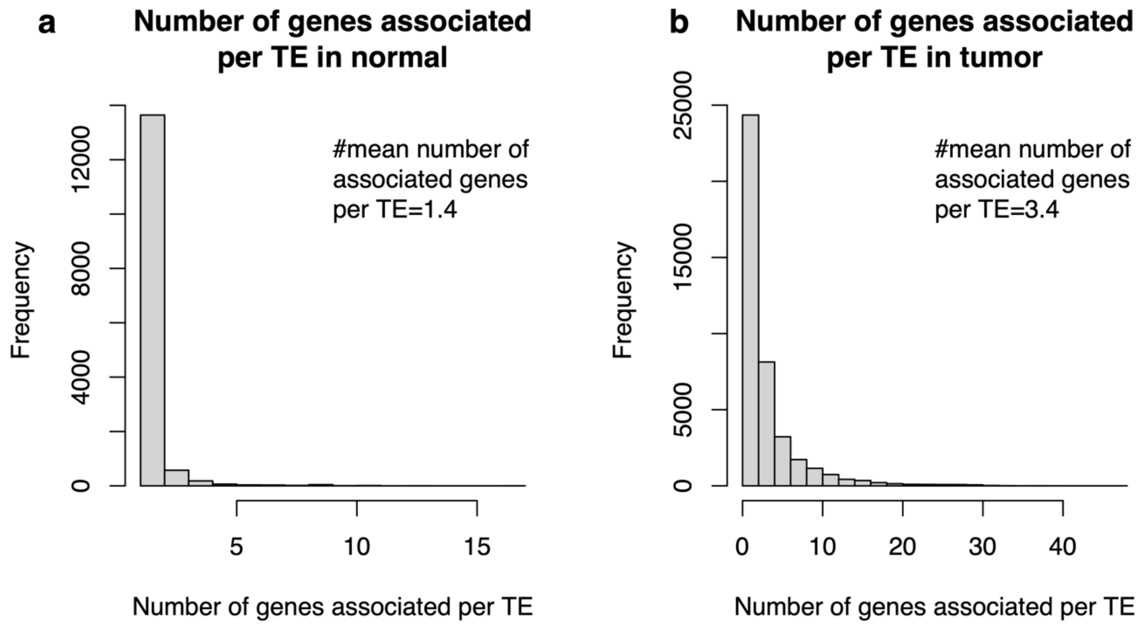

Supplementary figure 15 | Mean number of genes associated per TE in (A) normal and (B) tumor. We observe that in (**A**) normal there are less genes associated per TE (mean number of associated genes per TE = 1.4) compared to (**B**) tumor (mean number of associated genes per TE = 3.4). Source data are provided as a Source Data file.

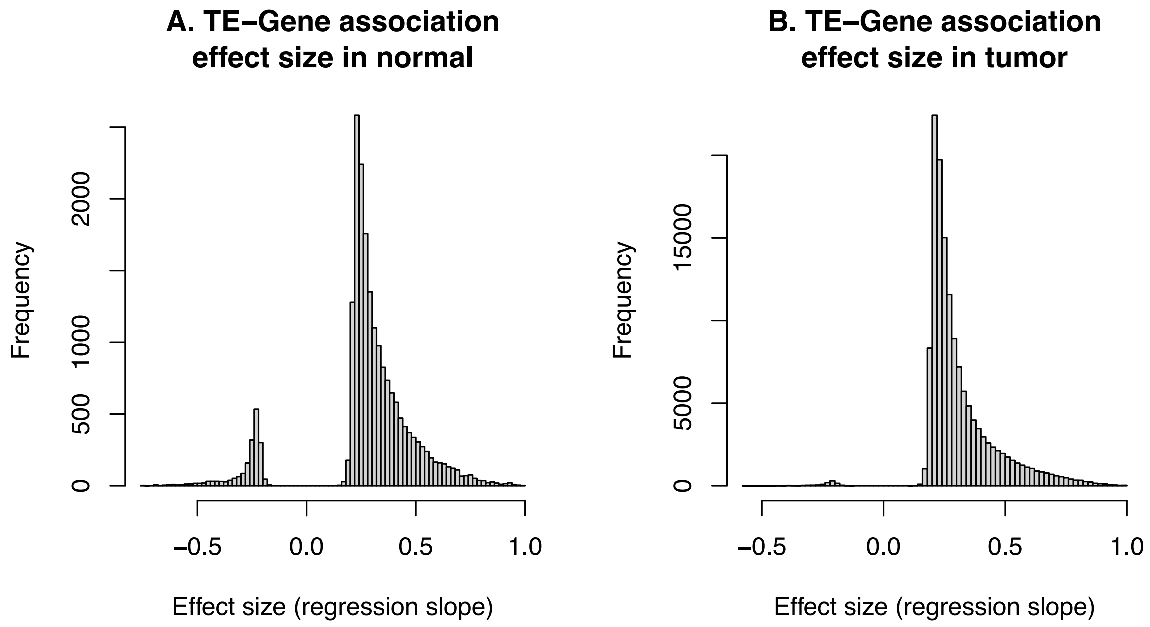

Supplementary figure 16 | TE-gene effect sizes (regression slope) in (A) normal and (B) tumor. We observe that most TEs are positively associated with a gene. Source data are provided as a Source Data file.

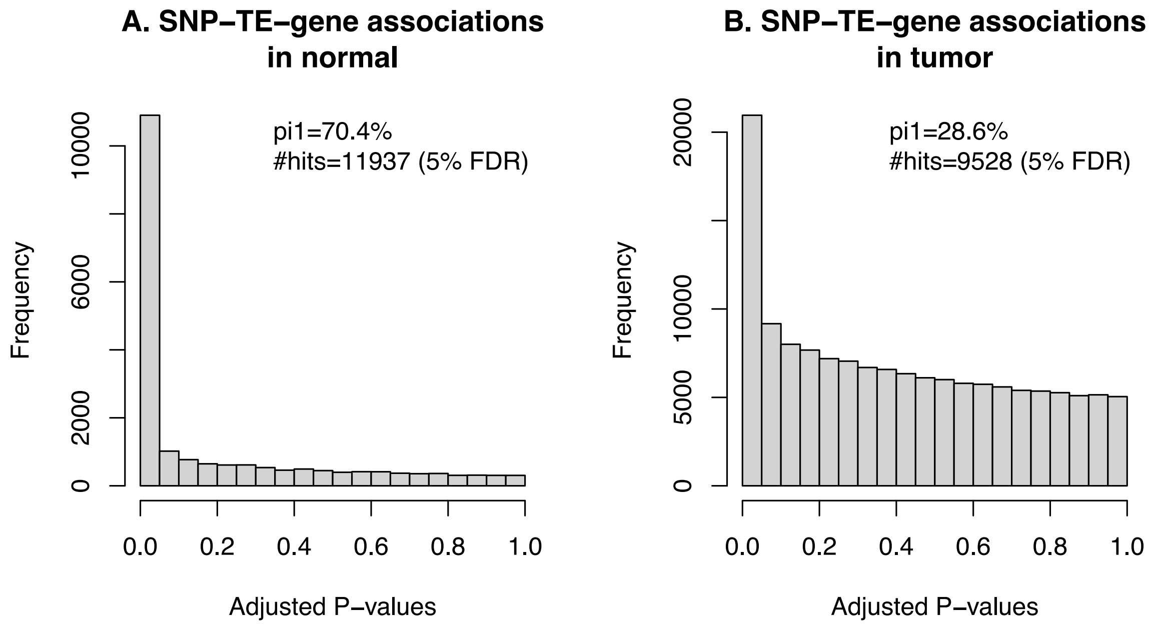

Supplementary figure 17 | P-value distribution of eQTL TE-gene discovery in (A) normal and (B) tumor. We observe that in normal (A) we discovered 11,937 triplets and in (B) tumor 9,528 triplets at 5% False Discovery Rate (FDR). Source data are provided as a Source Data file.

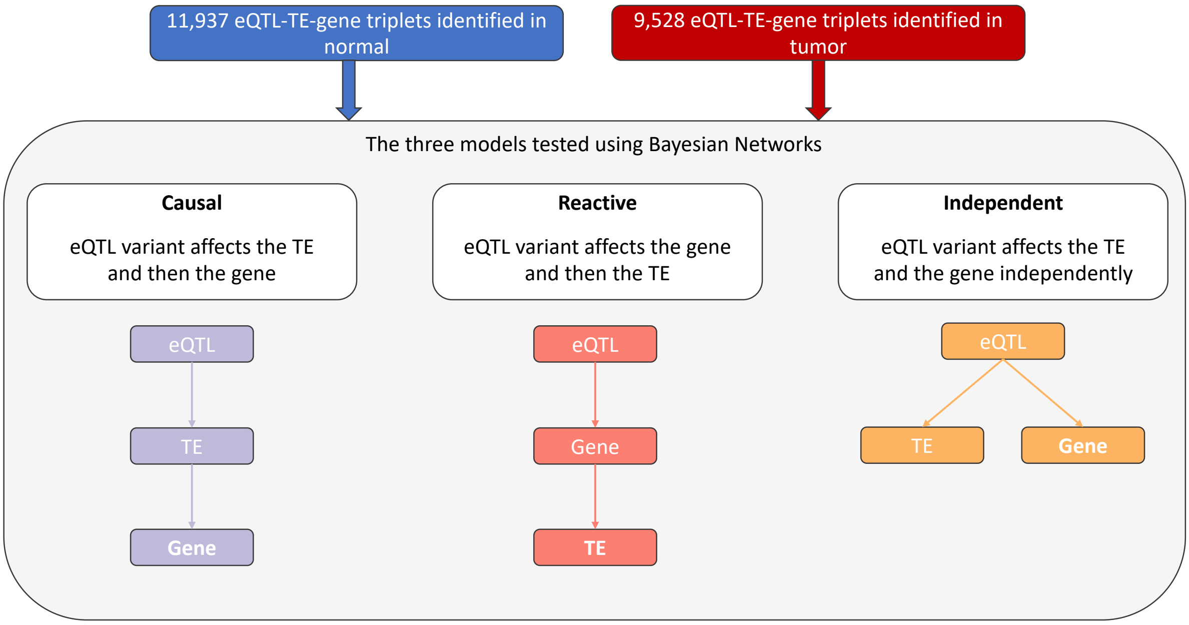

Supplementary figure 18 | Causal relationship of eQTLs, TEs and genes approach. To infer the most likely causal relationship between eQTL variants, TEs and genes, we tested three models using Bayesian Networks (BNs). The causal model where the eQTL variant affects the TE and then the gene, the Reactive model where the eQTL variants affects the gene and then the TE and the Independent model where the eQTL variant affects the TE and gene independently. For each triplet we obtained log likelihoods and we calculated posterior probabilities using a uniform prior probability for each of the three models.

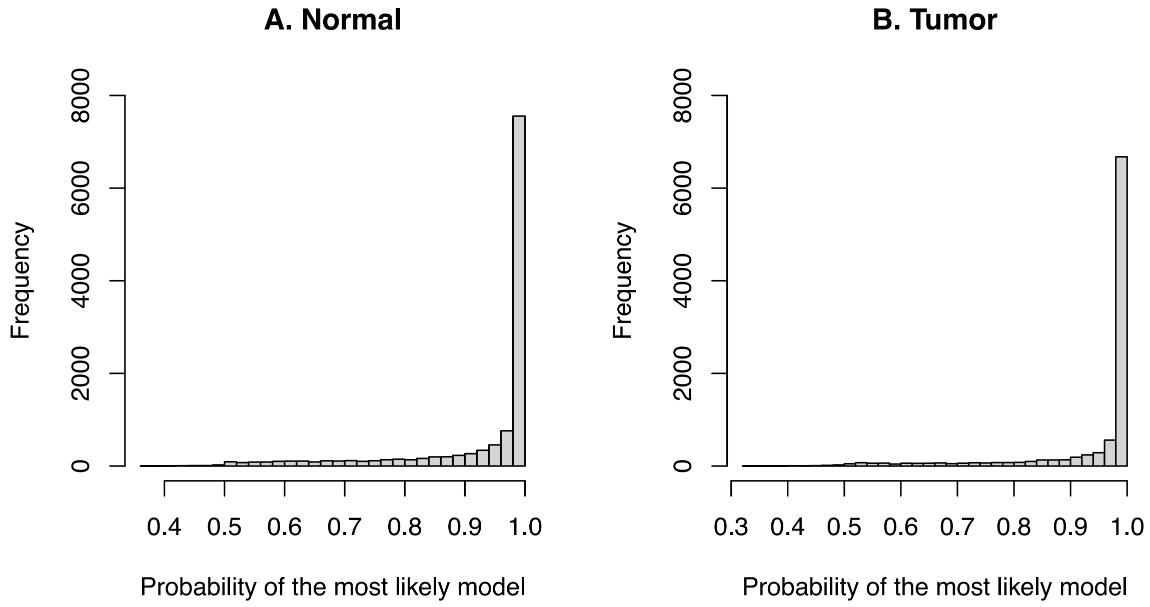

Supplementary figure 19 | Probability of the most likely model in (A) normal and (B) tumor. We can see that the probabilities of the most likely models are for most part above 0.8. Source data are provided as a Source Data file.

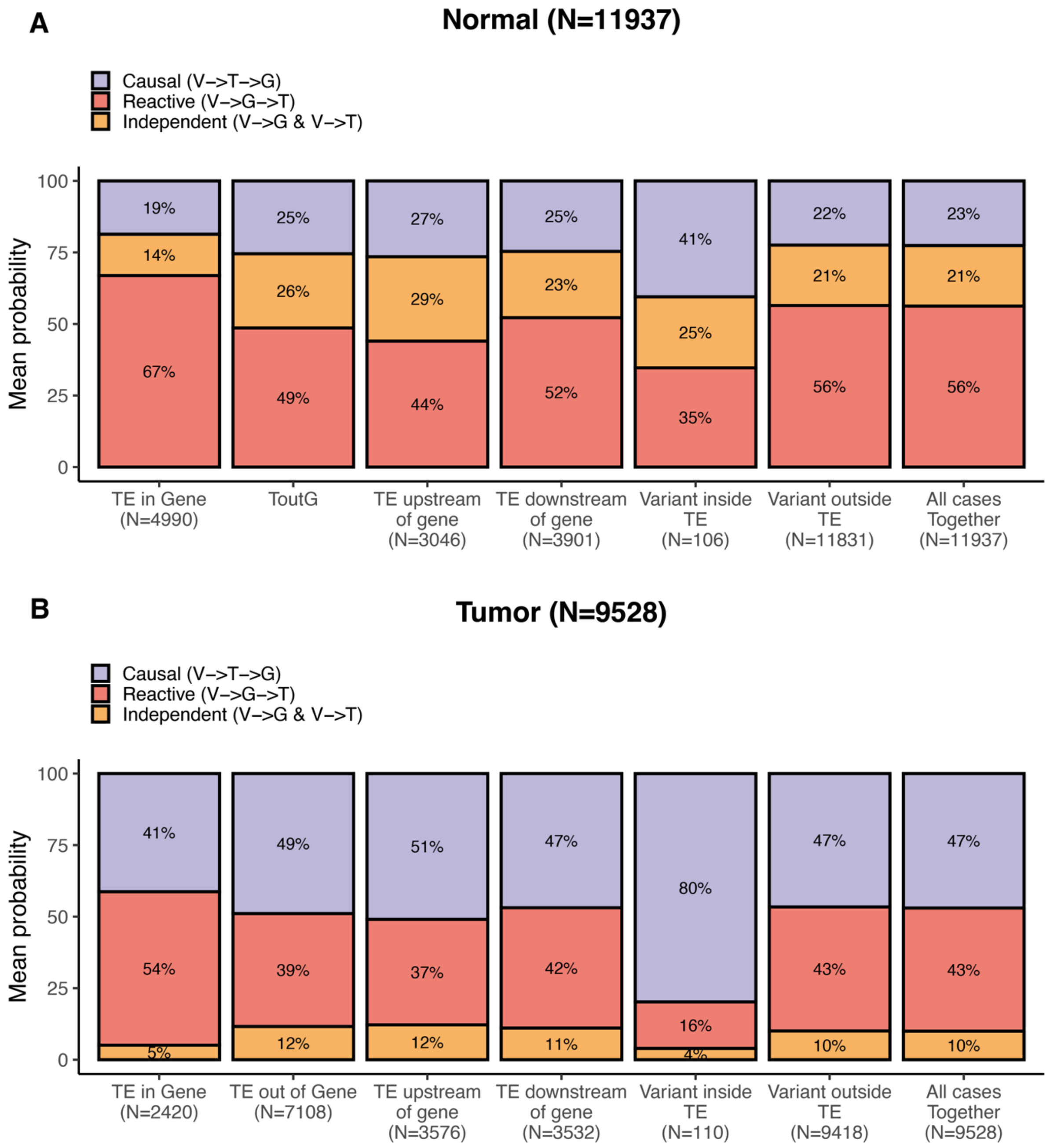

Supplementary figure 20 | Causal relationships depending on the genomic position of the TE in respect to the gene. (**A**) represents the causal relationships depending on the genomic position of the TE in respect to the gene in normal for the 11,937 triplets and (**B**) in tumor for the 9,528 triplets. In each case, we worked out the percentages by averaging the posteriors given by the Bayesian networks across all the triplets falling in each of the categories. We observe that TEs inside genes or downstream of genes tend to react to gene expression whereas TEs outside genes or when the eQTL variant is within the TE sequence, TEs are most likely causal for changes in gene expression. Source data are provided as a Source Data file.

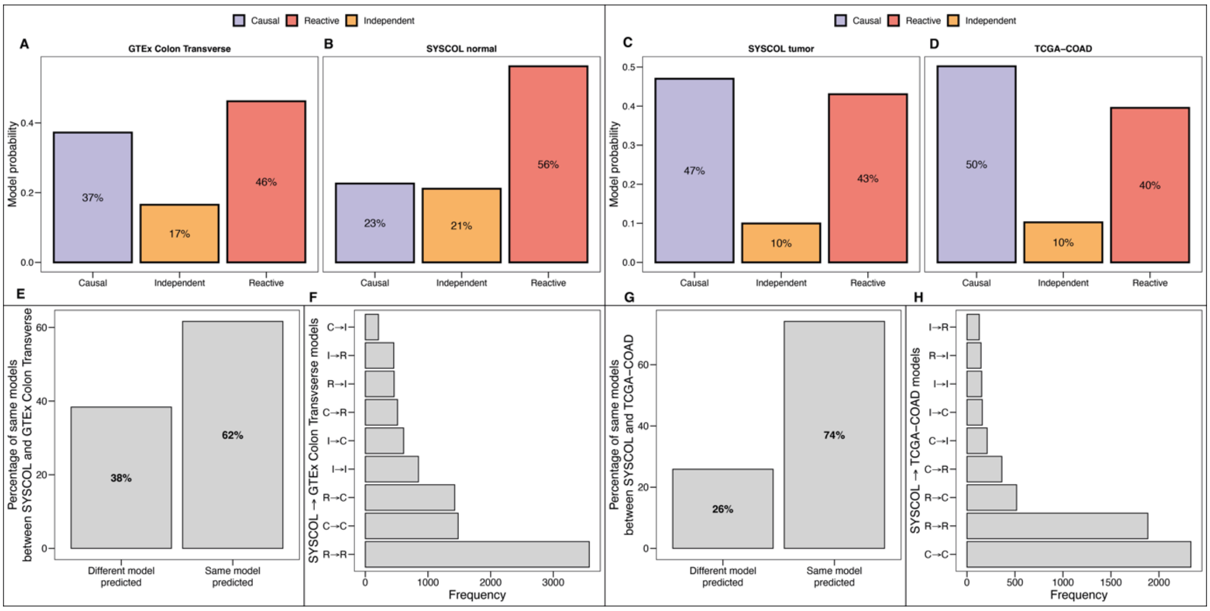

Supplementary figure 21: This figure represents the replication of the causal inference findings in external datasets. Model probabilities of the causal, reactive and independent models are represented **(A)** for the **9,577** SYSCOL normal triplets tested in GTEx colon transverse, **(B)** for the **12,379** normal triplets tested in SYSCOL, **(C)** for the **9,714** tumor triplets tested in SYSCOL, **(D)** for the **5,893** SYSCOL tumor triplets tested in TCGA-COAD. **(E)** represents the percentage of SYSCOL normal triplets with the same model predicted in SYSCOL and in GTEx colon transverse. **(F)** represents the frequency of substitutions between the normal triplets in SYSCOL and GTEx transverse. **(G)** represents the percentage of tumor triplets with the same model predicted in SYSCOL and in GTEx colon transverse. **(H)** represents the frequency of substitutions between the tumor triplets in SYSCOL and TCGA-COAD. We observe a high replication of our causal inference findings in both GTEx colon transverse and TCGA-COAD. Moreover, because the sample size of GTEx colon transverse is smaller (N=174) compared to the one of SYSCOL normal (N=275), we expected a smaller replication of our finding. We observe that in TCGA-COAD, the percentage of replication is higher as the TCGA-COAD and SYSCOL tumor dataset have almost the same sample size ($N_{\text{SYSCOL tumor}}$=276; $N_{\text{TCGA-COAD}}$ = 251), thus higher statistical power. Source data are provided as a Source Data file.

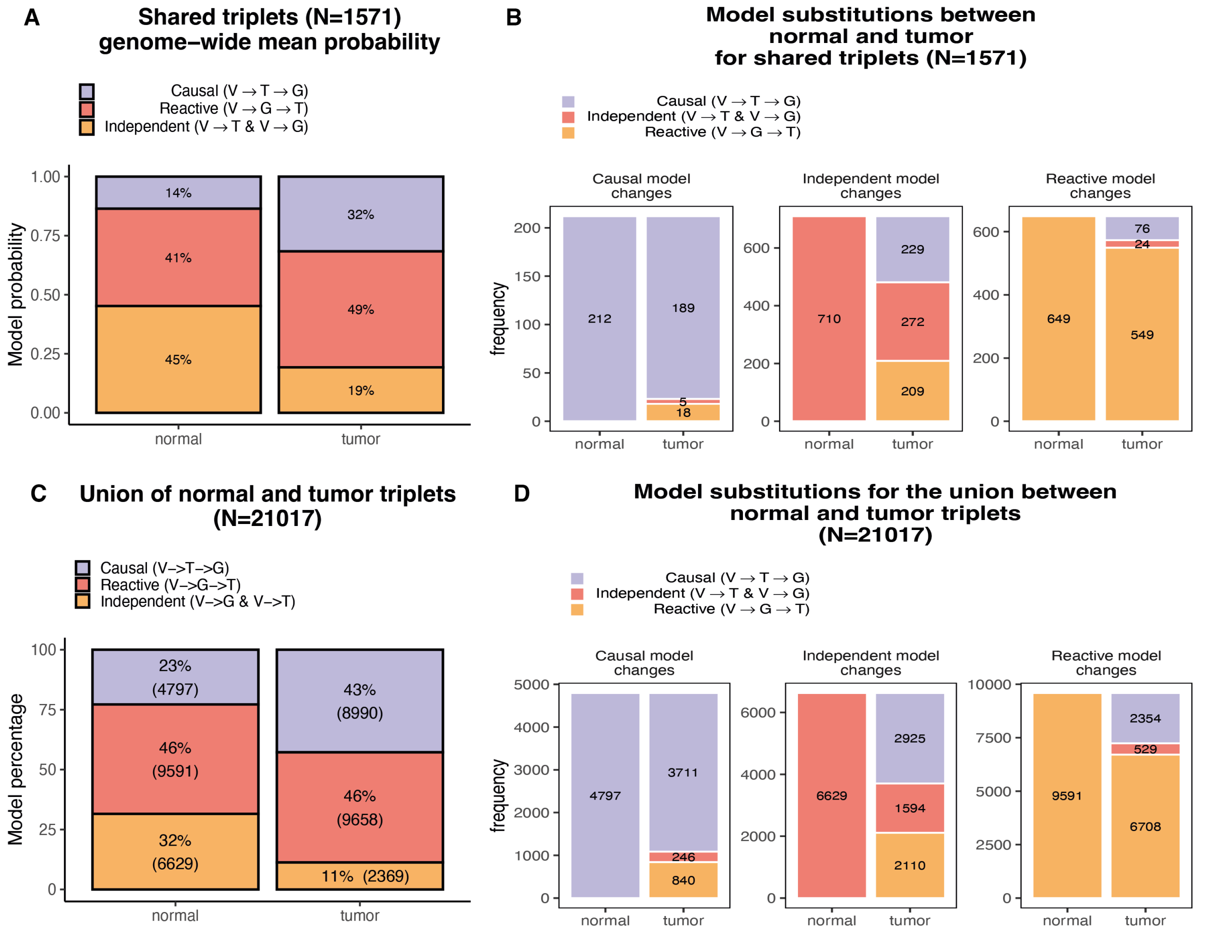

Supplementary figure 22 | Model shifts between normal and tumor. Model shifts for shared triplets between normal and tumor (A-B) where (A) represent the percentage of shared triplets tested that show either a higher posterior probability for the causal, reactive or independent model in tumor and normal. (**B**) Represents the model shifts from normal to tumor. (C-D) represent the same as A and B but for the union of normal and tumor triplets. In both shared and union, we observe an increase of the causal model in tumor. Independent models in normal and to a smaller extent reactive models are shifting for a causal model in tumor. Source data are provided as a Source Data file.

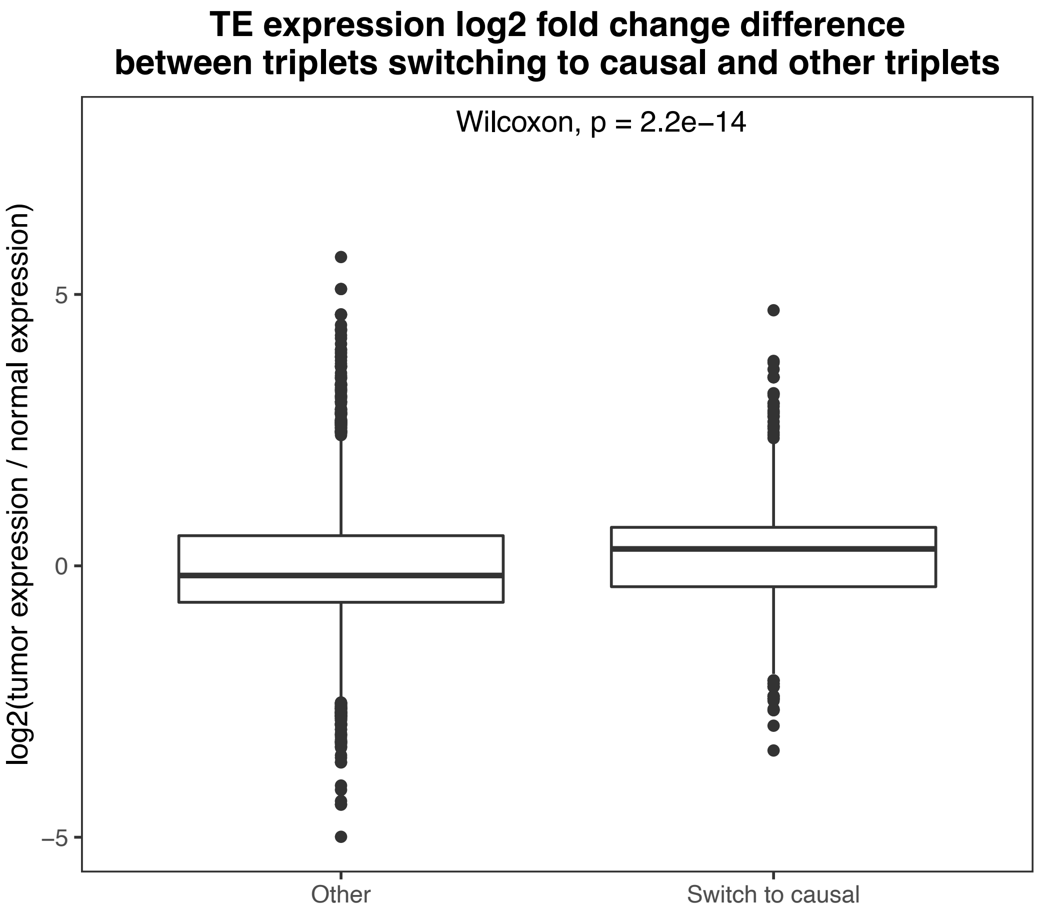

Supplementary figure 23 | log2 fold change of TEs switching to causal versus TEs that do not switch or switch but not to causal. We observe that TEs switching to causal in tumor (n=88) are significantly more upregulated compared to TEs not switching or switching but not to causal (n=4076) (two-sided Wilcoxon test, p = 2.2e-14), indicating that this upregulation could explain some of the cases where TEs switch to causal in tumor. Other boxplot values: minima = -4.992; 1st quartile = -0.671; median = -0.117; mean = -0.036; 3rd quartile = 0.555; maxima = 5.689. Switch to causal boxplot values: minima = -3.402; 1st quartile = -0.384; median = 0.312; mean = 0.2374; 3rd quartile = 0.709; maxima =4.71. Source data are provided as a Source Data file.
