# Supplementary material for: Transposable elements mediate genetic effects altering the expression of nearby genes in colorectal cancer
Source: Nat Commun. 2024 Jan 25;15:749. doi: 10.1038/s41467-023-42405-0 (PMC10811328; doi:10.1038/s41467-023-42405-0)
Supplement: Supplementary file 3 — Description of Additional Supplementary Files [file 41467_2023_42405_MOESM3_ESM.pdf]

Description of Additional Supplementary Files

Supplementary Data 1 - Enrichment analysis of expressed TEs compared to non-expressed TEs for regulatory regions. The data represents the enrichment analysis results of expressed TEs compared to non-expressed TEs for various regulatory regions. We observe that expressed TEs compared to non-expressed are enriched for all the different tested regulatory regions except for the enhancer regions.

Supplementary Data 2 - Discovered eQTLs at 5% False Discovery Rate (FDR) in normal. This represents the eQTL analysis results obtained by QTLtools for genes and TEs at 5% FDR in normal. We discovered a total of 10,231 TE and 6,955 gene-eQTLs in normal.

Supplementary Data 3 - Discovered eQTLs at 5% FDR in tumor. This represents the eQTLs analysis results obtained by QTLtools for genes and TEs at 5% FDR in tumor. We discovered a total of 5,199 TE- and 1,552 gene-eQTLs in tumor.

Supplementary Data 4 - Replication of SYSCOL normal eQTLs in GTEx colon transverse. This dataset represents the replication analysis results of normal eQTLs discovered in SYSCOL tested in GTEx colon transverse. Out of the 10,231 TE-eQTLs and 6,955 gene-eQTLs, 8,380 (82%) TE-eQTLs and 5,930 (85%) gene-eQTLs were present in GTEx colon transverse. We observed a high replication of our original results in normal with a pi1 of 0.831 for TE-eQTLs and pi1 of 0.686 for gene-eQTLs, corroborating our findings.

Supplementary Data 5 - Replication of SYSCOL tumor eQTLs in TCGA-COAD. This dataset represents the replication analysis results of tumor eQTLs discovered in SYSCOL tested in TCGA-COAD. Out of the 5,199 TE-eQTLs and 1,552 gene-eQTLs, 3,221 (62%) TE-eQTLs and 1,164 (75%) gene-eQTLs were present in TCGA-COAD. We observed a high replication of our original results in normal with a pi1 of 0.884 for TE-eQTLs and pi1 of 0.783 for gene-eQTLs, corroborating our findings.

Supplementary Data 6 - Shared eQTL discovery at 5% FDR. This dataset represents the analysis results of the shared eQTLs discovered at 5% FDR between normal and tumor. Shared-eQTLs are defined as eQTLs having the same effect on gene expression in both normal and tumor samples. We discovered a total of 525 shared TE-eQTLs and 175 shared gene-eQTLs.

Supplementary Data 7 - Tumor-specific eQTL discovery at 5% FDR. This dataset represents the analysis results of the tumor-specific eQTLs discovered at 5% FDR. Tumor-specific eQTLs are defined as eQTLs that have no effect on a particular TE or gene in normal but have an effect on the TE or gene expression in tumor. We discovered 429 tumor-specific TE-eQTLs and 117 tumor-specific gene-eQTLs.

Supplementary Data 8 - Functional enrichment of TE- and gene-eQTLs at 5% FDR in normal. This represents the functional enrichment analysis results of TE and gene-eQTLs at 5% FDR in normal. We used 231 ChIP-seq data from the Ensembl Regulatory Build to corroborate the biological relevance of

the TE- and gene-eQTLs discovered in normal. We observed that 5 of them have a higher enrichment for TE-eQTLs compared to gene-eQTLs.

Supplementary Data 9 - Functional enrichment of TE- and gene-eQTLs at 5% FDR in tumor. This represents the functional enrichments analysis results of TE and gene-eQTLs at 5% FDR in tumor. We used 231 ChIP-seq data from the Ensembl Regulatory Build to corroborate the biological relevance of the TE- and gene-eQTLs discovered in normal. We observed that 16 of them have a higher enrichment for TE-eQTLs compared to gene-eQTLs.

Supplementary Data 10 - Functional enrichment of tumor-specific and shared gene-eQTLs. This dataset represents the functional enrichment analysis results for the tumor-specific and shared gene-eQTLs. We used available ChIP-seq data from LoVo colorectal cancer cells for 220 TFs and 2 histone marks and observed that all tested TFs have a stronger enrichment for shared compared to tumor-specific gene-eQTLs.

Supplementary Data 11 - Functional enrichment of tumor-specific and shared TE-eQTLs. This dataset represents the functional enrichment results of tumor-specific and shared TE-eQTLs. We used available ChIP-seq data from LoVo colorectal cancer cells for 220 TFs and 2 histone marks and observed that all tested 58 TFs and 2 histone marks have a stronger enrichment for shared compared to tumor-specific TE-eQTLs pointing to tumor-specific TE regulation.

Supplementary Data 12 - Causal relationship of eQTL-TE-gene triplets in normal. This dataset represents the analysis results of the causal relationship discovered between eQTLs, TEs and genes in normal. We discovered a total of 11,937 eQTL-TE-gene triplets of which 2,702 are causal, 6,815 are reactive and 2,420 are independent.

Supplementary Data 13 - Causal relationship of eQTL-TE-gene triplets in tumor. This dataset represents the analysis results of the causal relationship discovered between eQTLs, TEs and genes in tumor. We discovered a total of 9,528 eQTL-TE-gene triplets of which 4,538 are causal, 4,156 are reactive and 834 are independent.

Supplementary Data 14 – Replication of normal eQTL-TE-gene triplets in GTEx colon transverse. This dataset represents the replication analysis results of the SYSCOL normal eQTL-TE-gene triplets tested in GTEx colon transverse. We discovered 9,577 eQTL-TE-gene triplets to be in common with the 11,937 triplets discovered in SYSCOL normal and observed a high replication of our findings with 62% similarity.

Supplementary Data 15 – Replication of tumor eQTL-TE-gene triplets in TCGA-COAD. This dataset represents the replication analysis results of the SYSCOL tumor eQTL-TE-gene triplets tested in TCGA-COAD. We discovered 5,893 eQTL-TE-gene triplets to be in common with the 9,528 triplets discovered in SYSCOL tumor and observed a high replication of our findings with 74% similarity.

Supplementary Data 16 – Causal relationship for shared eQTL-TE-gene triplets between normal and tumor. This dataset represents the shared eQTL-TE-gene triplets between normal and tumor. We discovered 1,571 shared eQTL-TE-gene triplets between normal and tumor.

Supplementary Data 17 - Causal relationship for the union of eQTL-TE-gene triplets between normal and tumor. This dataset represents the causal relationship of the union of eQTL-TE-gene triplets between normal and tumor. The union of eQTL-TE-gene triplets represents a total of 21,017 triplets.

Supplementary Data 18 – Hyperlinks and description of all the ChIP-seq experiments downloaded from ensembl ftp web page. The various hyperlinks can be used to directly download all compressed bed files used to generate the transcription factor and histone mark annotation used for the different functional enrichments of eQTLs analyses performed in the study.
