# Supplementary material for: Transposable elements mediate genetic effects altering the expression of nearby genes in colorectal cancer
Source: Nat Commun. 2024 Jan 25;15:749. doi: 10.1038/s41467-023-42405-0 (PMC10811328; doi:10.1038/s41467-023-42405-0)
Supplement: Supplementary file 5 — Reporting Summary [file 41467_2023_42405_MOESM5_ESM.pdf]

nature portfolio

Corresponding author(s): Nikolaos M.R. Lykoskoufis
Emmanouil T. Dermitzakis

Last updated by author(s): Aug 9, 2023

Reporting Summary

Nature Portfolio wishes to improve the reproducibility of the work that we publish. This form provides structure for consistency and transparency in reporting. For further information on Nature Portfolio policies, see our Editorial Policies and the Editorial Policy Checklist.

Statistics

For all statistical analyses, confirm that the following items are present in the figure legend, table legend, main text, or Methods section.

n/a	Confirmed	
☐	☒	The exact sample size (*n*) for each experimental group/condition, given as a discrete number and unit of measurement
☒	☐	A statement on whether measurements were taken from distinct samples or whether the same sample was measured repeatedly
☐	☒	The statistical test(s) used AND whether they are one- or two-sided<br>*Only common tests should be described solely by name; describe more complex techniques in the Methods section.*
☐	☒	A description of all covariates tested
☐	☒	A description of any assumptions or corrections, such as tests of normality and adjustment for multiple comparisons
☐	☒	A full description of the statistical parameters including central tendency (e.g. means) or other basic estimates (e.g. regression coefficient) AND variation (e.g. standard deviation) or associated estimates of uncertainty (e.g. confidence intervals)
☐	☒	For null hypothesis testing, the test statistic (e.g. *F*, *t*, *r*) with confidence intervals, effect sizes, degrees of freedom and *P* value noted<br>*Give P values as exact values whenever suitable.*
☐	☒	For Bayesian analysis, information on the choice of priors and Markov chain Monte Carlo settings
☒	☐	For hierarchical and complex designs, identification of the appropriate level for tests and full reporting of outcomes
☐	☒	Estimates of effect sizes (e.g. Cohen's *d*, Pearson's *r*), indicating how they were calculated

Our web collection on statistics for biologists contains articles on many of the points above.

Software and code

Policy information about availability of computer code

Data collection	No software was used for data collection.
Data analysis	We used QTLtools v1.3.1 (https://qtltools.github.io/qtltools/), clomics v.1.0 (https://github.com/odelaneau/clomics) and fenrichcpp v.1.0 (https://github.com/NLykoskoufis/fenrichcpp) for the various analyses of the study. Additionally, we used cutadapt v3.0 (https://cutadapt.readthedocs.io/en/stable/index.html), hisat2 v2.1.0 (http://daehwankimlab.github.io/hisat2/), BEDtools v2.30.0 (https://bedtools.readthedocs.io/en/latest/), birdseed2vcf software v1.2.2 (https://github.com/ding-lab/birdseed2vcf).

For manuscripts utilizing custom algorithms or software that are central to the research but not yet described in published literature, software must be made available to editors and reviewers. We strongly encourage code deposition in a community repository (e.g. GitHub). See the Nature Portfolio guidelines for submitting code & software for further information.

Data

Policy information about availability of data

All manuscripts must include a data availability statement. This statement should provide the following information, where applicable:

- Accession codes, unique identifiers, or web links for publicly available datasets
- A description of any restrictions on data availability
- For clinical datasets or third party data, please ensure that the statement adheres to our policy

All data generated during the current study are available in Supplementary Data 1-17. Supplementary Data 1-17 contains results obtained from the various analysis

performed using raw publicly available datasets. The RNA-sequencing data and genotype arrays for the 275 normal colon and 276 colorectal cancer samples from SYSCOL is available in the European Genome-Phenome Archive (EGA) under accession code EGAC00001000204 [https://ega-archive.org/dacs/EGAC00001000204]. This is restricted data and access can be requested through EGA. The microarray based DNA methylation data from the SYSCOL project is available in EGA under accession code EGAD00010001888 [https://ega-archive.org/datasets/EGAD00010001888]. The RNA-sequencing and germline genotypes from the GTEx dataset can be obtained through dbGAP under accession code phs000424.v8.p2 [https://www.ncbi.nlm.nih.gov/projects/gap/cgi-bin/study.cgi?study_id=phs000424.v8.p2]. The RNA-sequencing and germline genotypes from TCGA database can be obtained through dbGAP under accession code phs000178.v11.p8 [https://www.ncbi.nlm.nih.gov/projects/gap/cgi-bin/study.cgi?study_id=phs000178.v11.p8]. Both GTEx and TCGA datasets are restricted data and access can be requested through dbGAP. Colorectal cancer LoVo cell line ChIP-seq data can be obtained from Gene Expression Omnibus (GEO) under accession code GSE49402 [https://www.ncbi.nlm.nih.gov/geo/query/acc.cgi?acc=GSE49402]. Transcription factors and histone marks ChIP-seq data can be downloaded from the Ensembl FTP site [http://ftp.ensembl.org/pub/grch37/release-100/regulation/homo_sapiens/Peaks/] where we downloaded all compressed bed files for all cell types. The full list of hyperlinks of all ChIP-seq datasets from Ensembl used in the current study can be found in Supplementary Dataset 18.

Human research participants

Policy information about studies involving human research participants and Sex and Gender in Research.

Reporting on sex and gender	not applicable
Population characteristics	not applicable
Recruitment	not applicable
Ethics oversight	not applicable

Note that full information on the approval of the study protocol must also be provided in the manuscript.

Field-specific reporting

Please select the one below that is the best fit for your research. If you are not sure, read the appropriate sections before making your selection.

☒ Life sciences ☐ Behavioural & social sciences ☐ Ecological, evolutionary & environmental sciences

For a reference copy of the document with all sections, see nature.com/documents/nr-reporting-summary-flat.pdf

Life sciences study design

All studies must disclose on these points even when the disclosure is negative.

Sample size	We used a total of 275 normal samples and 276 tumor samples. This sample size was used because it gave sufficient statistical power for cis-eQTL discovery
Data exclusions	No data were excluded from this analysis
Replication	All softwares used for this study are publicly availble. We used GTEx data to replicate and corroborate our findings.
Randomization	Randomization was not used in this study since there are no experimental groups
Blinding	Blinding is not relevant to this study since no group allocation occurs.

Reporting for specific materials, systems and methods

We require information from authors about some types of materials, experimental systems and methods used in many studies. Here, indicate whether each material, system or method listed is relevant to your study. If you are not sure if a list item applies to your research, read the appropriate section before selecting a response.

Materials & experimental systems

n/a	Involved in the study
☒	☐ Antibodies
☒	☐ Eukaryotic cell lines
☒	☐ Palaeontology and archaeology
☒	☐ Animals and other organisms
☒	☐ Clinical data
☒	☐ Dual use research of concern

Methods

n/a	Involved in the study
☒	☐ ChIP-seq
☒	☐ Flow cytometry
☒	☐ MRI-based neuroimaging
